# The effects of altered BMP4 signaling in first branchial-arch-derived murine embryonic orofacial tissues

**DOI:** 10.1038/s41368-021-00142-4

**Published:** 2021-11-29

**Authors:** Jue Xu, Meiling Chen, Yanan Yan, Qiaoxue Zhao, Meiying Shao, Zhen Huang

**Affiliations:** 1 West China School of Public Health and Department of Stomatology, West China Fourth Hospital, Sichuan University, Chengdu, China; 2 Southern Center for Biomedical Research and Fujian Key Laboratory of Developmental and Neuro Biology, College of Life Sciences, Fujian Normal University, Fuzhou, China

**Keywords:** Cell lineage, Cell proliferation

## Abstract

The first branchial arch (BA1), which is derived from cranial neural crest (CNC) cells, gives rise to various orofacial tissues. Cre mice are widely used for the determination of CNC and exploration of gene functions in orofacial development. However, there is a lack of Cre mice specifically marked BA1’s cells. *Pax2*-Cre allele was previously generated and has been widely used in the field of inner ear development. Here, by compounding *Pax2*-Cre and *R26R-*mTmG mice, we found a specific expression pattern of *Pax2*^+^ cells that marked BA1’s mesenchymal cells and the BA1-derivatives. Compared to *Pax2*-Cre; *R26R*-mTmG allele, GFP^+^ cells were abundantly found both in BA1 and second branchial arch in *Wnt1*-Cre;*R26R*-mTmG mice. As BMP4 signaling is required for orofacial development, we over-activated *Bmp4* by using *Pax2*-Cre; *pMes*-BMP4 strain. Interestingly, our results showed bilateral hyperplasia between the upper and lower teeth. We also compare the phenotypes of *Wnt1*-Cre; *pMes*-BMP4 and *Pax2*-Cre; *pMes*-BMP4 strains and found severe deformation of molar buds, palate, and maxilla-mandibular bony structures in *Wnt1*-Cre; *pMes*-BMP4 mice; however, the morphology of these orofacial organs were comparable between controls and *Pax2*-Cre; *pMes*-BMP4 mice except for bilateral hyperplastic tissues. We further explore the properties of the hyperplastic tissue and found it is not derived from *Runx2*^+^ cells but expresses Msx1, and probably caused by abnormal cell proliferation and altered expression pattern of p-Smad1/5/8. In sum, our findings suggest altering BMP4 signaling in BA1-specific cell lineage may lead to unique phenotypes in orofacial regions, further hinting that *Pax2*-Cre mice could be a new model for genetic manipulation of BA1-derived organogenesis in the orofacial region.

## Introduction

The normal embryogenesis and morphogenesis of cranial-facial organs such as frontonasal, midbrain, craniofacial skeleton, eyes, nose, lip, palate, jaw, teeth, and external and inner ears depend on the appropriate migration and differentiation of cranial neural crest (CNC) cells^1,2^. During cranial-facial development, CNC cells migrate to their ventral-lateral destination and form branchial arches (BAs). Specifically, the first branchial arch (BA1) is further divided into maxillary and mandibular prominences and is essential for the morphogenesis of tissues in maxilla-mandibular regions. Consequently, BA1 differentiated into characteristic orofacial tissues, such as teeth, dentary bone, and secondary palate^3,4^.

Cre mice are widely used in inducible systems for studying the functions of a specific gene. In the field of orofacial organogenesis, transgenic mice were used to trace the lineage-dependent CNC cells. *Wnt1*-Cre and *P0*-Cre are extensively used in the study of craniofacial development^5–8^. Specifically, as *Wnt1* promoter activated in neural crest pre- and post-migratory progenitors, *Wnt1*-Cre mice were accepted as the most widely used allele in studying the patterning of craniofacial organs^5,9,10^. However, the *Wnt1*^+^ or *P0*^+^ cells existed not only in BA1 but also widely distributed in the second BA (BA2)^10,11^. Although some other specific Cre mice are also used for studying the development of orofacial organs or tissues, they are restricted to a certain subgroup of CNC cells. *Osr2*-Cre mice were generated and used for tissue-specific studies in palate development^11–13^. But the *Osr2*-Cre is limited to study the palatogenesis and pattern formation during palate development because of the specific expression of *Osr2* in palatal shelves. *Dmp1*-Cre is also an allele that can be used in the craniofacial region; however, the *Dmp1*^+^ cells were restricted in osteocytes in maxilla-mandibular regions^14,15^. *Osx*-Cre mice is another strain that has been used for studying the mechanisms of both bone and dental mesenchymal tissue^16,17^. Therefore, it is difficult to study the specific BA1-derived orofacial organs or tissues by using the above Cre mice.

The bone morphogenetic proteins (BMPs) are morphogens that are essential for pattern formation of neurogenesis, ossification, organogenesis, and the pre- and post-natal tissue growth^18^. In orofacial regions, BMP signaling is necessary for organs or tissues’ development including palate, teeth, skeletons, and temporomandibular joint (TMJ)^19^. BMPs, BMP receptor Ia (BMPRIa), and BMP antagonist are expressed in the palate and regulate palatogenesis of mice^20–24^. In teeth development, BMP signaling plays critical roles in initiation, morphogenesis, mineralization, and root formation^19,25^. Lack or over-activation of BMP signaling in either tooth’s epithelial or mesenchymal cells leads to malformation of tooth^21,26–28^. The skeletogenesis in cranial-facial including the skull formation as well as mandibular development are regulated by BMP signaling^29–32^. Members of BMP signaling such as BmprIa and Bmp2 also exert biological functions during the development of TMJ^33,34^. Among these, Bmp4 expresses in maxillary and mandibular processes of BA1 and contributes to the development of orofacial organs such as tooth, palate, maxilla, and mandible^19^. However, the researchers all selected the above-mentioned traditional Cre mice (*Wnt1*-Cre etc.) to explore the mechanisms of BMP4 signaling in the orofacial region^21,27,32,35^. According to the limitations of the Cre distribution of these mouse strains, the exact influences of BMP4 signaling in orofacial organs or tissues may probably be hidden.

In this study, we took advantage of *Pax2*-Cre^36^ and *R26R-*mTmG mice^37^ to trace the lineage of *Pax2*^+^ cells in the oral-facial region and found these cells were specifically expressed in BA1 while the *Wnt1*^+^ cells were both detected in BA1 and BA2. Thus, the Pax2-Cre had immense potential as a transgenic mouse model for the lineage tracing of BA1-derived mesenchymal cells. Subsequently, we use *Pax2*-Cre mice to detect the function of BMP4 signaling in BA1-derived orofacial mesenchyme by using *pMes*-Bmp4 allele^32^. We also compared the craniofacial phenotype of *Pax2*-Cre; *pMes*-BMP4 and *Wnt1*-Cre; *pMes*-BMP4 mice. Our results reveal the specific effects of BMP4 signaling in BA1-derived murine embryonic orofacial regions.

## Results

### *Pax2*^+^ cell lineage was specifically expressed in BA1 and the derivatives

Ohyama and colleagues have generated *Pax2*-Cre mice and further successfully used *Pax2*-Cre mice in their studies focused on the development of inner ear^36,38^. Here we first compounded *Pax2*-Cre and *R26R-*mTmG mice to determine the expression of *Pax2* and to trace *Pax2*^+^ lineage cells in the developing orofacial regions.

**Fig. 1:**
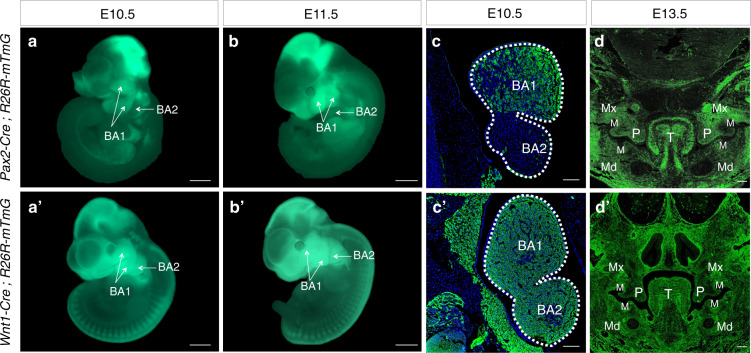
The expression pattern of *Pax2*^+^ and *Wnt1*^+^ cell lineages. **a**, **bʹ** *Pax2*^+^ CNC cells, evidenced by GFP expression, were specifically detected in BA1 of *Pax2*-Cre; *R26R*-mTmG embryos at E10.5 (**a**) and E11.5 (**b**), as compared to the expression of GFP in both BA1 and BA2 of *Wnt1*-Cre; *R26R*-mTmG mice at E10.5 (**aʹ**) and E11.5 (**bʹ**). **c**, **dʹ** Immunofluorescence staining showed *Pax2*^+^ cell linage was distributed in BA1’s mesenchyme (**c**) and the derivatives in orofacial regions (**d**); however, *Wnt1*^+^ cells were found both in BA1 and BA2 (**cʹ**), and the derived tissues in craniofacial regions (**dʹ**). BA1 the first branchial arch, BA2 the second branchial arch, T, tongue; P, palatal shelf; Mx, Maxilla; Md, mandible; M, molar bud. Scale bars = 500 μm (**a**, **bʹ**); Scale bars = 100 μm (**c**, **dʹ**)

Live imaging on *Pax2*-Cre; *R26R-*mTmG embryos revealed *Pax2*^+^ lineage cells (labeled as GFP^+^ cells) existed specifically in BA1 except for the head region (Fig. 1a, b) while the GFP^+^ cells of *Wnt1*-Cre; *R26R-*mTmG mice were found not only in midbrain but also in BA1 and BA2 from E10.5 through E11.5 (Fig. 1aʹ, bʹ). An intensive distribution of *Pax2*^+^ and *Wnt1*^+^ cell lineages in the BAs was observed by anti-GFP staining at E10.5 (Fig. 1c, cʹ). GFP-positive cells were limited in mesenchyme of BA1 and barely found in BA2 in *Pax2*-Cre; *R26R-*mTmG mice (Fig. 1c). On the other hand, *Wnt1*^+^ lineage cells were found both in BA1 and BA2 mesenchyme (Fig. 1cʹ). The *Pax2*^+^ and *Wnt1*^+^ cells were detected in mesenchyme of orofacial organs (Fig. 1d, dʹ).

### Forced *Bmp4* in BA1-derived orofacial regions leads to hyperplasia between upper and lower jaws

To evaluate the effects of BMP4 signaling in BA1-derived orofacial regions, we used *Pax2*-Cre allele to locally activate *Bmp4* by compounding the Pax2-Cre allele with *pMes*-BMP4 allele.

**Fig. 2:**
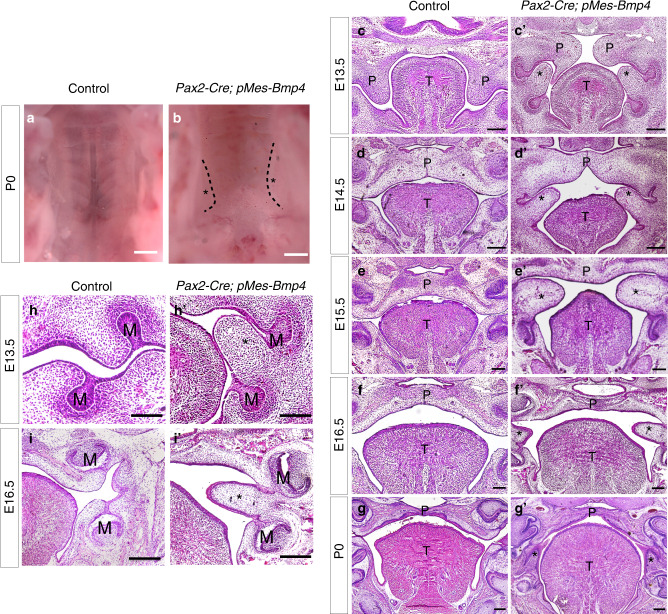
Enhanced BMP4 signaling results in abnormal hyperplastic soft tissue in orofacial regions. **a**, **b** Whole-mount view of palate in control (**a**) and *Pax2*-Cre; *pMes*-BMP4 mice (**b**) at P0. The mutant shows bilateral hyperplasia (**b** asterisks marked). **c**–**gʹ** Coronal sections of E13.5-P0 mice of controls’ and mutants’ heads reveal the abnormal bilateral hyperplastic soft tissues located between upper and lower molar buds (**cʹ**–**gʹ**, asterisks marked). **h**, **iʹ** The growth direction of maxilla-mandibular molar buds was changed because of hyperplasia (**hʹ, iʹ** asterisks marked). T, tongue; P, palate; M, molar bud. Scale bars = 500 μm (**aʹ**, **bʹ**); Scale bars = 100 μm (**c**–**iʹ**)

We found the *Pax2*-Cre; *pMes*-BMP4 mice died soon after birth, and compared to the control, the stomach of the mutant lack of milk (data not shown). But the mutant mice did not exhibit cleft palate defect, which is one of the most common birth defect of mice. Instead, the oral view of the whole-mount unraveled a unique palatal abnormity: the bilateral soft tissue hyperplasia located between upper and lower jaws (Fig. 2b, marked by asterisk). Histological analysis of *Pax2*-Cre; *pMes*-BMP4 mice showed the existence of hyperplastic soft tissue as early as E13.5 (Fig. 2cʹ). Specifically, we found the abnormal tissue extended from the region between maxillary and mandibular molars and consists of epithelial and mesenchymal cells. In addition, the size of the hyperplastic soft tissue gradually increased from E13.5 to E15.5, and then decreased after E15.5 (Fig. 2cʹ–gʹ). Although the growth direction of tooth buds was changed because of the hyperplasia, the size morphology was comparable to the controls (Fig. 2h, iʹ). Although the morphology of tongue in mutants seem different from the controls, the MF20 marked muscle were comparable to the controls (Supplemental Fig. 1).

### *Wnt1*-Cre; *pMes*-BMP4 and *Pax2*-Cre; *pMes*-BMP4 mutant mice exhibit different phenotypes in orofacial organs

**Fig. 3:**
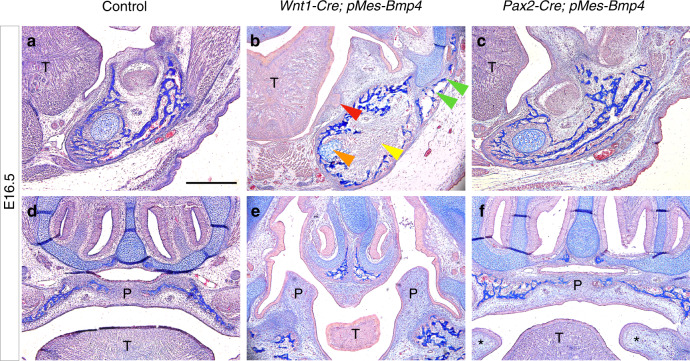
Coronal sections revealed structures of molar buds, palate and mandible of control, *Wnt1Cre*; *pMes*-Bmp4 and *Pax2*-Cre; *pMes*-BMP4 head between the mandible and maxilla at E16.5. **a**–**c** The morphology of molar buds was distinctly defected in *Wnt1Cre*; *pMes*-Bmp4 mice (**b**, red arrowhead), but there is no obvious change between the *Pax2*-Cre; *pMes*-BMP4 (**c**) and control mice (**a**). Bony syngnathia was showed in *Wnt1Cre*; *pMes*-Bmp4 mice (**b** green arrowheads). The mandibular bony structures (yellow arrowhead) and Meckel’s cartilage (orange arrowhead) were greatly inhibited in *Wnt1Cre*; *pMes*-Bmp4 mice while these structures were comparable between *Pax2*-Cre; *pMes*-BMP4 (**c**) and control mice (**a**). **d**–**f** The palatal shelves were fused in control (**d**) and *Pax2*-Cre; *pMes*-BMP4 mice (**f**); however, cleft palate was showed in *Wnt1Cre*; *pMes*-Bmp4 mice (**e**). Specifically, bilateral hyperplastic soft tissues (asterisks marked) were existed between maxilla-mandibular molar buds in Pax2-Cre; pM*es*-BMP4 mice. Scale bar = 500 µm

To give a clear picture of orofacial bone and tooth phenotypes in CNC-derived cells, we compare the tooth buds, mandibular bones, Meckel’s cartilage, and palate between *Wnt1Cre*; *pMes*-Bmp4 and *Pax2*-Cre; *pMes*-BMP4 heads (Fig. 3). In our results, the molar buds, mandibular bony structures, and Meckel’s cartilage were severely destructed in *Wnt1Cre*; *pMes*-Bmp4 mice (Fig. 3b); however, the morphology of the above organs showed barely difference between *Pax2*-Cre; *pMes*-BMP4 mice and controls (Fig. 3c). Notably, bony fusion between the upper and lower jaws specifically existed in *Wnt1Cre*; *pMes*-Bmp4 mice (Fig. 3b, green arrowheads). In control and *Pax2*-Cre; *pMes*-BMP4 mice, bilateral palatal shelves were successfully fused aside the tongue laterally at E16.5; however, cleft palate existed in *Wnt1Cre*; *pMes*-Bmp4 mice and the palatal shelves showed a reversed direction (the tip pointing dorsally to the nasal septum) (Fig. 3d–f). In *Pax2*-Cre; *pMes*-BMP4 mice, although there is no cleft palate, the abnormal hyperplastic soft tissues was found between maxilla-mandibular jaws (Fig. 3f, asterisks marked).

### Overexpression of BMP4 signaling alters cellular behavior and gene expression in orofacial regions

To explore the properties of the hyperplastic soft tissue, we first detected the expression of Msx1, the critical gene of palate and teeth during development. In controls, Msx1 expressed in mesenchymal cells of tooth germ and anterior palate but not in middle and posterior parts of palatal shelf (Fig. 3a–c). In mutants, the expression level and pattern of Msx1 in palatal shelf were comparable to the controls; however, Msx1 expression in tooth buds slightly decreased and the *Msx1*^+^ cells were detected in mesenchyme of hyperplastic soft tissue from anterior to posterior part (Fig. 3aʹ–cʹ). We also detect the osteogenic differentiation marker, Runx2, in orofacial regions of *Pax2*-Cre; *pMes*-BMP4 mice and the controls at E15.5. We found Runx2 expressed in the ossification areas of palate, maxilla, and mandible from anterior to posterior parts both in controls and mutants and the expression levels are comparable (Fig. 3d–fʹ). However, Runx2^+^ cells were absent in the hyperplastic tissue (Fig. 3dʹ–fʹ).

**Fig. 4:**
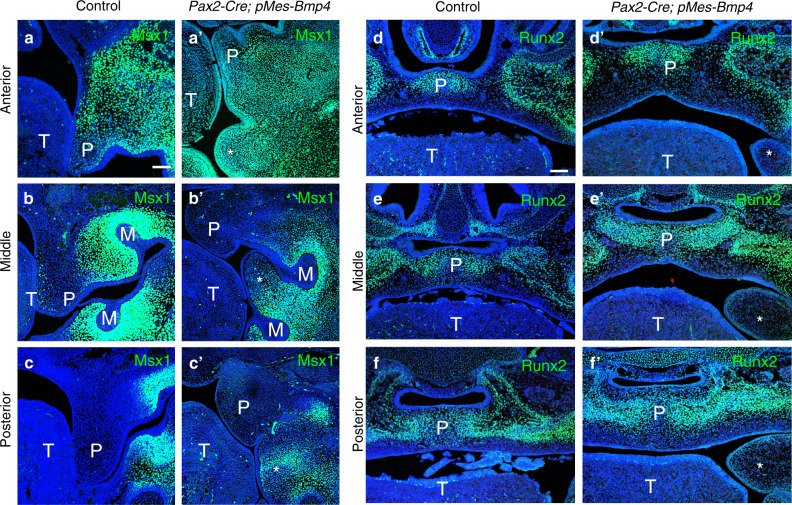
Altered expression of orofacial landscape genes in *Pax2*-Cre; *pMes*-BMP4 mice. **a**–**cʹ** Immunostaining with anti-Msx1 antibody reveals the expression of Msx1 in anterior palatal shelves and mesenchyme of tooth buds in both controls and mutants (**a**, **bʹ**), and the mesenchymal cells of the abnormal hyperplasia (**aʹ**–**cʹ**, asterisks marked). **d**–**fʹ** Runx2 were detected in ossification regions in maxilla-mandibular regions in both controls and mutants (**d**–**fʹ**) but no expression was found in hyperplastic tissues in *Pax2*-Cre; *pMes*-BMP4 mice (**dʹ**–**fʹ**, asterisks marked) at E15.5. T, tongue; P, palate; M, molar bud. Scale bars = 100 μm (**a**–**fʹ**)

Because the hyperplastic soft tissue exists in the orofacial region of *Pax2*-Cre; *pMes*-BMP4 mice, we used Ki67 antibody to conduct a cell proliferation assay at E11.5 before the hyperplasia phenotype became recognizable. In the results, the Ki67^+^ cells were statistically significantly increased in *Pax2*-Cre; *pMes*-BMP4 mice (Fig. 4; *P* < 0.05). However, the levels of cell apoptosis in the mutants were comparable to the controls (Supplemental Fig. 2).

**Fig. 5:**
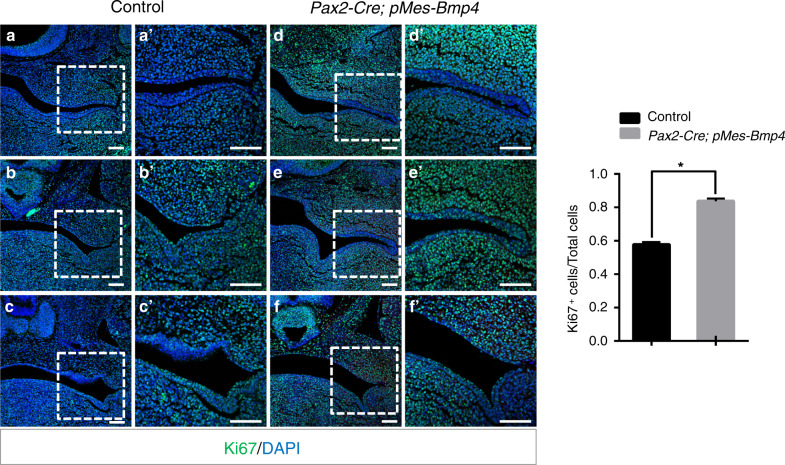
*Pax2*-Cre-driven Bmp4 expression causes abundant cell proliferation in *Pax2*-Cre; *pMes*-BMP4 mice. Ki67-positive cells in three different maxilla-mandibular regions (**a**–**fʹ**) reveal an abundant proliferation rate in *Pax2*-Cre; *pMes*-BMP4 mice compared to the control mice (**g** *P < 0.05). T, tongue; P, palate; M, molar bud. Scale bars = 100 μm (**a**–**fʹ**)

**Fig. 6:**
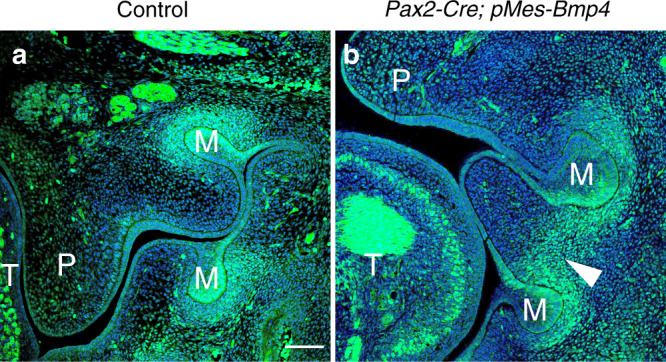
The distributions of pSmad1/5/8 in the *Pax2*-Cre; *pMes*-BMP4 (**b**) and control mice (**a**) at E13.5. T, tongue; P, palate; M, molar bud. Scale bars = 100 μm (**a**, **b**)

To explore the underlying mechanism of hyperplastic soft tissues, phosphorylated Smad1/5/8 (pSmad1/5/8) was also detected. There is no difference for expression of pSmad1/5/8 in palatal shelves between mutants and controls (Supplemental Fig. 3). However, pSmad1/5/8 specifically expressed in the basal part of the hyperplasia between upper and lower molar buds (Fig. 5b, arrowheads, Fig. 6).

## Discussion

Orofacial organs or tissues were originated from BA1-specific CNCs. In the field of orofacial development, researchers usually choose Cre transgenic mice to study the organogenesis or developing processes of the orofacial organs or tissues. However, there is no BA1-specific Cre mice currently. Therefore, to study the development of orofacial organs which are originated from BA1, we better use an allele that has specific CNC cell labeling in BA1. In this study, we utilized *Pax2*-Cre; *R26R-*mTmG mice to trace the CNC-derived *Pax2*^+^ cells in the developing orofacial region and explore the specific effects of BMP4 signaling in these BA1 derivatives.

Pax2 is a transcriptional factor that is widely expressed during development in mammals. In the craniofacial region, *Pax2* was found to appear in the developing otocyst and is considered as the marker of the otic placode^39^. Thus, *Pax2*-Cre mice were generated widely and used in the researches of inner ear development^36,40^. In our results, we surprisingly found *Pax2* promoter drives expression in the neural crest post-migratory progenitors, resulting in permanent Cre-mediated GFP reporter activity in BA1 and there is barely GFP^+^ cell in BA2. On the other hand, the GFP^+^ cells existed both in BA1 and BA2 of *Wnt1*-Cre; *R26R-*mTmG mice. Therefore, the *Pax2*^+^ cell lineage has a more specific expression pattern in CNC-derived BA1. The expression in skeletogenic mesenchyme of teeth, palate, upper and lower jaws, and tongue also confirmed that *Pax2*^+^ cell lineages contribute to BA1-derived orofacial organs during embryonic development in mice. One previous study using *Pax2*-Cre mice to conditionally knockout *Dicer1* results in cleft palate^41^ supports the feasibility of *Pax2*-Cre allele in the field of orofacial organs’ development.

It is acceptable that BMP4-mediated signaling is required for normal craniofacial development including teeth formation and palatogenesis^21,42,43^. Lack of *Bmp4* in CNC cells results in abnormal size and morphology of teeth as well as some percentage of cleft palate^21^. On the other hand, over-activation of BMP4 signaling in CNC lineage by using *Wnt1*-Cre mice demonstrated a specific craniofacial abnormity, congenital bony syngnathia, which manifested as bony fusion between maxilla and mandible, hypoplastic mandible, and cleft palate^32^. In our study, we investigated the effects of gain-of-function of BMP4 signaling by using BA1-specific *Pax2*-Cre transgenic mice. Surprisingly, the *Pax2*-Cre; *pMes*-BMP4 mice exhibit quite different orofacial phenotype, compared to the *Wnt1*-Cre; *pMes*-BMP4 mice. The severe defects of tooth, maxilla-mandibular bones, and complete cleft palate were shown in *Wnt1*-Cre; *pMes*-BMP4 mice; the results were comparable as previous study^32^. On the other hand, only the hyperplasia between upper and lower jaws was found in *Pax2*-Cre; *pMes*-BMP4 mice in our study. It may suggest that the wide distribution of *Wnt1*^+^ cells leads to severe craniofacial phenotypes, but the BA1-derived *Pax2*^+^ cell lineage has a limited distribution in orofacial regions and results in mild influences in orofacial organs.

To explore the influences on gene expression in orofacial regions and to confirm the properties of the hyperplastic tissue in *Pax2*-Cre; *pMes*-BMP4 mice, we detected the expression level of Msx1 and Runx2. Msx1 normally expresses in mesenchymal cells of tooth bud as well as anterior palate, but not in posterior palate^20^. Here we found the expression levels of Msx1 in anterior palate and teeth were comparable but Msx1 expresses in the mesenchyme of hyperplastic tissue from anterior to posterior parts in *Pax2*-Cre; *pMes*-BMP4 mice. Moreover, the hyperplastic tissue is located between upper and lower molar buds. It suggests this abnormal tissue is much closer to the tooth mesenchyme than palatal shelf. Hard tissues, including maxilla, mandible, hard palate, and the majority of teeth, are common but essential for morphogenesis and function in orofacial regions. Besides, in *Wnt1*-Cre; *pMes*-BMP4 mice, osteogenic differentiation was significantly inhibited^32^. So, we also detected the expression of Runx2 in *Pax2*-Cre; *pMes*-BMP4 mice. Runx2 expression in palate as well as the mesenchymal cells of tooth buds was comparable in mutants and controls; however, we cannot detect Runx2-positive cells in hyperplastic tissue of the mutant mice. This demonstrates that the hyperplasia does not fall into bony tissues.

Considering cell proliferation and apoptosis might be closely related to the phenotype of hyperplasia, we separately tested the rate of proliferation and apoptosis between mutants and controls. In our results, cell proliferation was significantly increased while cell apoptosis is comparable to the control group. It is consistent with the existence of hyperplastic soft tissue between upper and lower jaws in *Pax2*-Cre; *pMes*-BMP4 mice. However, these results are totally the opposite in *Wnt1*-Cre; *pMes*-BMP4 mice: there is no difference in cell proliferation rate between mutants and the control mice, but the abundant ectopic apoptosis in *Wnt1*-Cre; *pMes*-BMP4 mice. Therefore, cell behaviors driven by BMP4-related signaling might be decided by a different source of CNC cells and the hyperplasia is caused by abnormal abundance of cell proliferation.

**Fig. 7:**
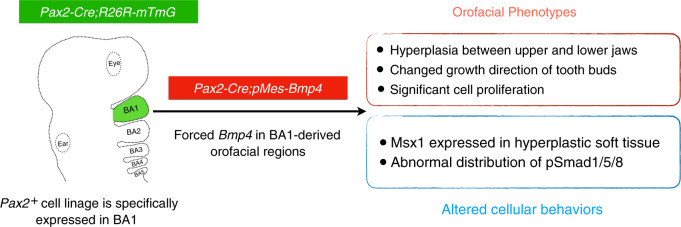
Schematic representations of specific cell lineage of Pax2-positive cells in BA1 as well as the effects of enhanced BMP4 signaling in Pax2^+^ lineage cells. BA the branchial arch

In BMP4-mediated signaling, pSmad1/5/8 is an essential factor that can bind to Smad4, then enters the nucleus and interact with transcription factors to regulate downstream gene expression in orofacial organs such as palates and teeth^13,21,28,44^. Here, we showed a specific expression pattern of pSmad1/5/8 between upper and lower molar buds and the basal part of the hyperplastic soft tissue in *Pax2*-Cre; *pMes*-BMP4 mice. It suggests the hyperplasia might be associated with the abnormal activation of pSmad1/5/8 in mesenchymal cells between upper and lower molar buds. Based on our results, we summarize the specific cell lineage of *Pax2*^*+*^ and the effects of BMP4 signaling in Pax2-labeled BA1-derived mesenchymal cells in Fig. 7.

In conclusion, the present study indicates the importance of the *Pax2*-Cre mice to achieve specific neural crest cell labeling in BA1 and can be an optional tool for future studies during the development of orofacial organs. We also demonstrate the specific effects of BMP4 regulated signaling in BA1-derived mesenchyme compared to the *Wnt1*-Cre; *pMes*-BMP4 mice: enhanced BMP4 signaling leads to bilateral hyperplasia between upper and lower molar buds that is possibly caused by the significant increase of cell proliferation as well as the abnormal distribution of pSmad1/5/8 in the orofacial regions. Hence, our results further demonstrate the balanced BMP4 signaling is essential for the normal patterning and development in BA1-derived orofacial organs.

## Materials and methods

### Animals

*Pax2*-Cre, *R26R-*mTmG, *pMes*-Bmp4, and *pMes*-Noggin transgenic mice used in this study have been previously described^24,32,36,37^. Staged *Pax2*-Cre; *R26R*-mTmG, *Pax2*-Cre; *pMes*-Bmp4 and *Pax2*-Cre; *pMes*-Noggin embryos or newborns were collected from timed pregnant females for further experiments. The usage of animals and the experiments were approved by the Institutional Animal Care and Use Committee of Fujian Normal University (Approval NO. IACUC-20200010).

### Histological analysis

The embryonic heads from E13.5 through P0 were fixed in 4% paraformaldehyde at 4 °C overnight. Then the samples were dehydrated in gradient alcohols. Subsequently, the samples were embedded with paraffin and sectioned according to the routine method. The 8 µm slices were then subjected to hematoxylene and eosin (H&E) staining or Azon red/Aniline blue staining as described^32^.

### Immunofluorescent staining

For immunofluorescent staining, the sliced samples were performed according to the manufacturer’s instructions. The primary antibodies used were as follows: anti-GFP (Abcam, 1:1 000), anti-Ki67 (Abcam, 1:500), anti-Msx1 (Abcam, 1:500), anti-Runx2 (Santa Cruz, 1:300), anti-pSmad1/5/8 (Millipore, 1:500), anti-MF20 (Invitrogen, 1:200), type II Collagen (1:500, Abcam, ab34712), α-SMA (Invitrogen, 1:200). Alexa Fluor 488 (Thermo Fisher Scientific, 1:1 000) was used as secondary antibody. The tissues were then viewed under a confocal fluorescence microscope (Carl Zeiss, Göttingen, Germany). All the experiments in this study were repeated at least three times.

### TUNEL analysis

The cell apoptosis was analyzed by using TUNEL assay following the use of the manufacturer’s instruction of FragEL DNA Fragmentation Detection Kit, Fluorescein (Calbiochem, Darmstadt, Germany).

### Statistical analysis

All the experiments and analyses were repeated at least three times. We randomly selected three different parts of maxilla-mandibular regions of the same size from at least three different samples and the number of the Ki67-positive (Ki67^+^) cells and apoptotic cells were counted by using ImageJ (version 1.46r, National Institutes of Haealth) and were analyzed by the Student’s *t* test. ^*^P < 0.05 was regarded to be statistically significant. The results were presented as the means with standard deviation.

## Supplementary information

Supplemental data
