# Supplementary material for: The effects of altered BMP4 signaling in first branchial-arch-derived murine embryonic orofacial tissues
Source: Int J Oral Sci. 2021 Nov 29;13:40. doi: 10.1038/s41368-021-00142-4 (PMC8630201; doi:10.1038/s41368-021-00142-4)
Supplement: Supplementary file 1 — Supplemental data [file 41368_2021_142_MOESM1_ESM.docx]

**The effects of altered BMP4 signaling in first branchial arch derived murine embryonic orofacial tissues**

**Running title: BMP4 signaling in BA1-derived orofacial tissues**

**Authors:**

Jue Xu^1^, Meiling Chen^2^, Yanan Yan^2^, Qiaoxue Zhao^2^, Meiying Shao^1*^, Zhen Huang^2*^

**Affiliations:**

^1^West China School of Public Health, and Department of Stomatology, West China Fourth Hospital, Sichuan University, Chengdu, 610041 Sichuan, P.R. China

^2^Southern Center for Biomedical Research and Fujian Key Laboratory of Developmental and Neuro Biology, College of Life Sciences, Fujian Normal University, Fuzhou, 350117 Fujian, China

**
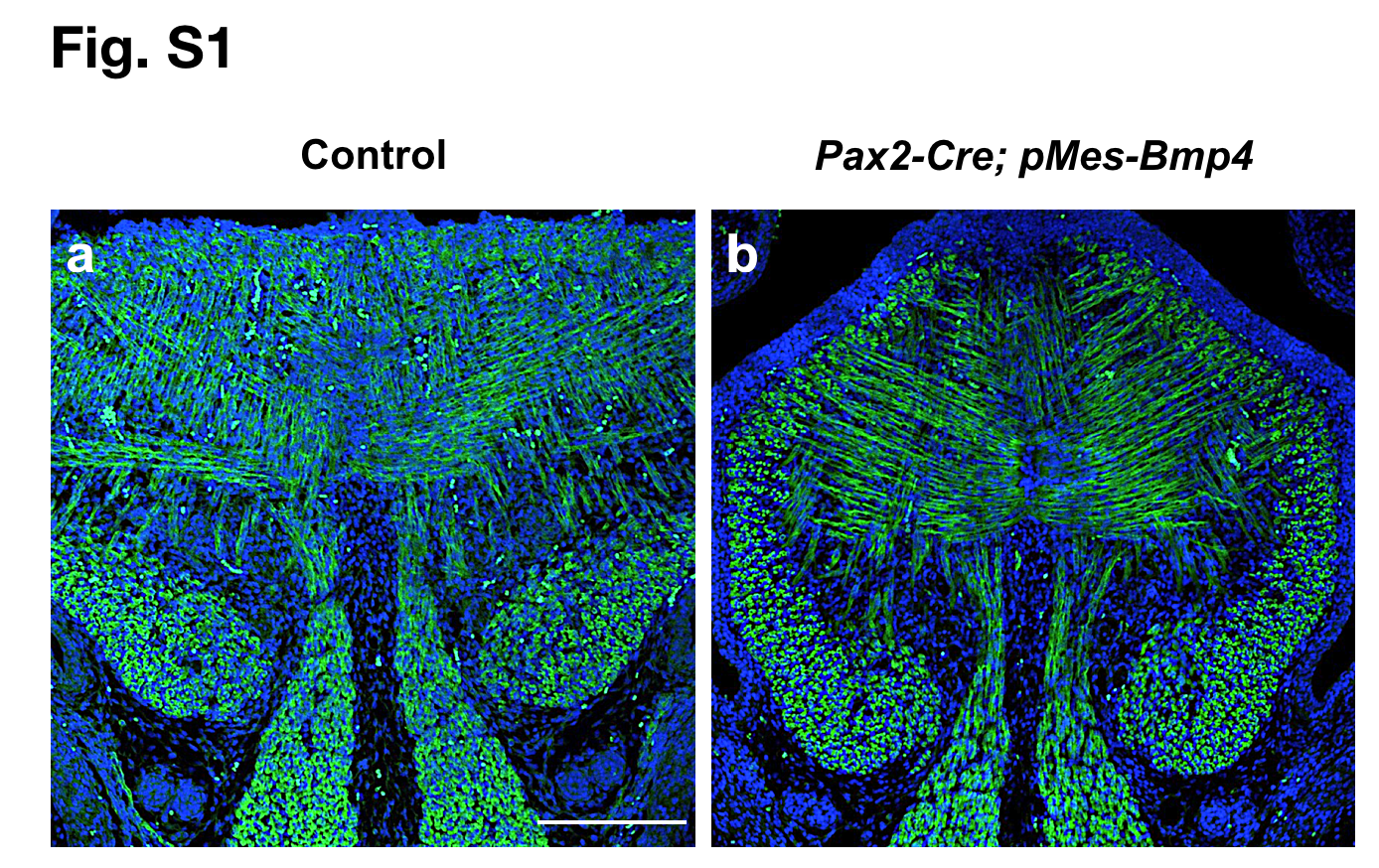
**

**Supplemental Figure 1** Immunostaining showed the patterning of the intrinsic and extrinsic muscles (marked by MF20) in the E13.5 tongue of *Pax2*-Cre; *pMes*-BMP4 (**b**) and control mice (**a**) were comparable. Scale bar= 100 μm.


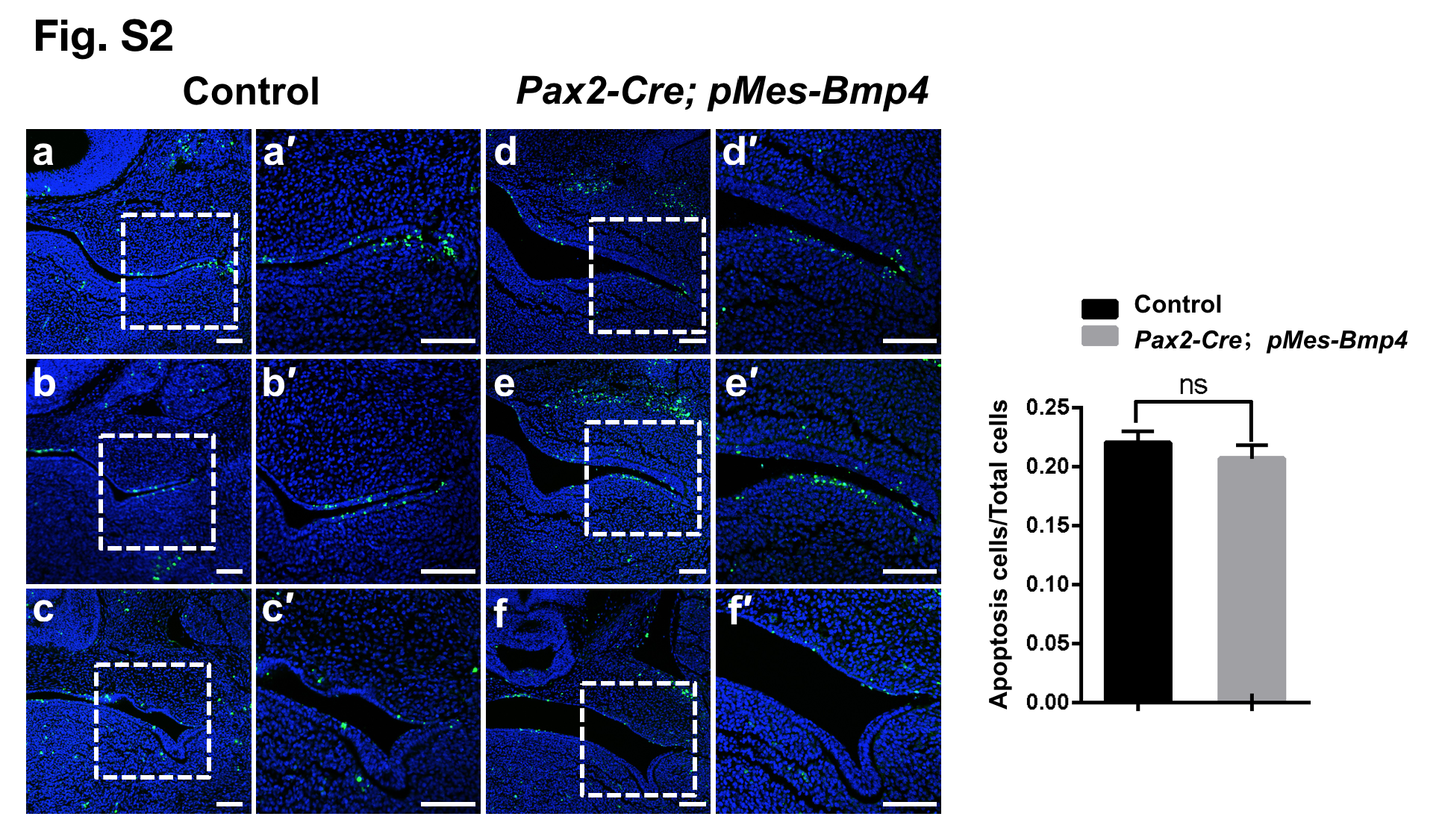


**Supplemental Figure 2** Tunel assay indicated there’s no statistical difference of apoptotic cells between mutant mice (**d-f’**) and controls (**a-c’**). Scale bars= 100 μm (**a-f’**).

**
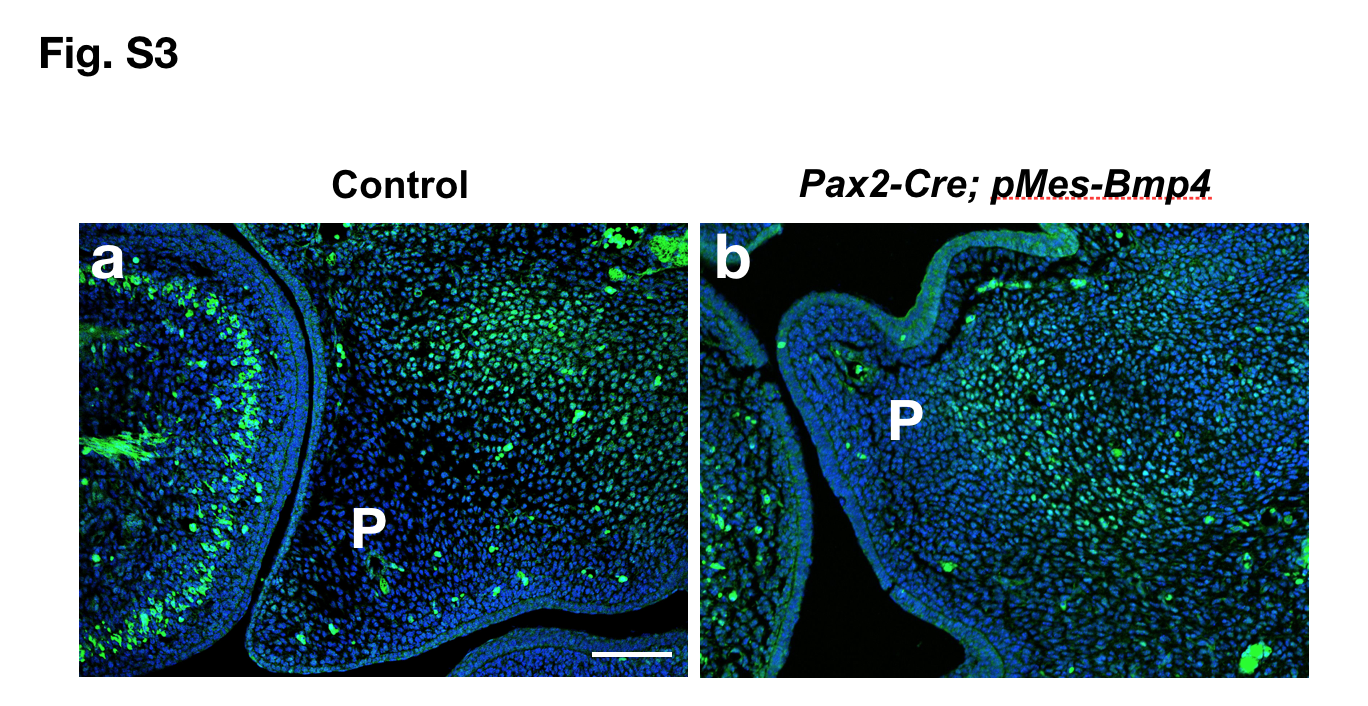
**

**Supplemental Figure 3** The distributions of pSmad1/5/8 in palate were comparable in *Pax2*-Cre; *pMes*-BMP4 (**b**) and control mice (**a**) at E13.5. P, palate. Scale bar= 100 μm.
